# Relation of Teeth, Tonsils and Intestinal Toxemias to Diseases of the Eye*Read before the Section on Ophthalmology at the Seventieth Annual Session of the American Medical Association, Atlantic City, N. J., June, 1919. Because of lack of space, this article is abbreviated in *The Journal*. The complete article appears in the Transactions of the Section and in the author’s reprints. A copy of the latter will be sent by the author or by *The Journal* on receipt of a stamped addressed envelope.

**Published:** 1919-11

**Authors:** George Huston Bell

**Affiliations:** Attending Surgeon, New York Eye and Ear Infirmary (Eye Department) New York


### RELATION OF TEETH, TONSILS AND INTESTINAL TOXEMIAS TO DISEASES OF THE EYE *

BY GEORGE HUSTON BELL, M.D.
Attending Surgeon, New York Eye and Ear Infirmary (Eye Department) New York

The presence and fatality of diseases whose essence is toxemia make them a subject of prime importance to the whole medical profession. I have selected the subject of focal infections for this paper, for the reason that it is a field in which I have long been interested, having read a paper [1] on this subject before the New York Institute of Stomatology, at the New York Academy of Medicine, in October, 1910. My belief has grown stronger and stronger that there is not sufficient attention paid to focal infections as a real cause of diseases of the eye. The epoch-making work of Rosenow [2] on the transmutation and selective localization of pyogenic organisms in the various tis-

---

* Read before the Section on Ophthalmology at the Seventieth Annual Session of the American Medical Association, Atlantic City, N. J., June, 1919. Because of lack of space, this article is abbreviated in *The Journal.* The complete article appears in the Transactions of the Section and in the author's reprints. A copy of the latter will be sent by the author or by *The Journal* on receipt of a stamped addressed envelope.

1 Bell, G. H.: Ocular Manifestations of the Peripheral Affections of the Fifth Cranial Nerve, M. Rec. 79: 863 (May) 1911.

2 Rosenow, E. C.: Iritis and Other Ocular Lesions: on Intravenous Injection of Streptococci, J. Infect. Dis. 17: 403, 1915.

sues of the body has revolutionized some of our ideas of etiology and pathology. The experimental and clinical investigations of Brown and Irons [3] have been especially valuable to us, and we know now that the eye may suffer from such infections, as well as joints, muscles, nerves and blood vessels. Wescott,[4] Haskins,[5] Harrison [6] and others have recently presented interesting articles in this connection.

What I now wish to do is to bring to your attention the grouping of the most potent focal infections which we encounter in the practice of ophthalmology. For the lack of a better classification I call them the "three T's," which stand for teeth, tonsils and toxemia of the intestinal tract.

Every patient who comes to my office must stand the "acid test" of the three T's, and the same routine is carried out, as far as possible, in my clinic at the New York Eye and Ear Infirmary. It takes only a few minutes to subject patients to this examination, provided there is a laboratory man at hand, yet how often it is overlooked and forgotten. If one has the three T's well in mind constantly, there is no chance to overlook any of the great sources of focal infections. The ramifications of the three T's are so interwoven that it is impossible for me to dissociate them. In thinking of one in connection with a patient, the other two always come into my mind. In other words, they go hand in hand. This paper is written with the understanding that, when necessary, syphilis, gonorrhea and an occasional sinus or tuberculosis trouble must be eliminated or excluded.

---

[3] Brown and Irons: The Aetiology of Iritis, Tr. Amer. Oph. Soc. 1916, p. 495.

[4] Wescott, C. D.: Inflammations of the Eye and Focal Infections, Railway S. J. 22: 457, 1916.

[5] Haskins, W. H.: Diseases of Eye in Focal Infections of the Teeth, Dental Cosmos 58: 1039, 1916.

[6] Harrison, W. G.: Probable Role of Teeth and Tonsils in the Etiology of Inflammatory Eye Diseases, Am. J. Ophth. 1: 660 (Sept.) 1918.

THE TEETH

Much has already been written and much more can be written about dental infections as a cause of diseases of the eyes. Too much stress can not be laid on a thorough examination of the teeth, which includes: (1) an inspection of the mouth; (2) palpation of the gums, and (3) roentgenograms of all the teeth, dead or alive, pivots, arches and bridges.

Barker[7] calls attention to the fact that "serious arthritis, osteitis, osteomyelitis, periostitis, myositis, embolic pneumonia, pleuritis, endocarditis, neuritis, neuralgia, iritis, anemia, septicemia and pyemia may, in certain cases, have their origin in oral sepsis." And I believe there is a growing tendency to look on the same etiologic factor as contributory in many instances of chronic degenerative diseases like arteriosclerosis and arterial hypertension.

Case 1.—A man, aged 22, came to my clinic at the New York Eye and Ear Infirmary, July 15, 1917, with plastic iritis in both eyes. When first seen, both pupils were contracted and the aqueous was cloudy. There was severe pain in both eyes, considerable exudation in the left pupillary space, and marked ciliary tenderness and injection. The patient was ordered into the hospital at once and had the usual treatment for iritis. The Wassermann test was + + + +. Inunctions were started. As the teeth were badly decayed, fourteen of them were extracted during his first ten days in the hospital. Improvement was so rapid that I decided to discontinue the inunctions. The patient made a rapid recovery, leaving the hospital at the end of four weeks. He had gained 14 pounds in weight in the nine weeks that elapsed after he had the teeth extracted. His vision is now normal in both eyes. He was given seven inunctions while in the hospital. I proposed to treat him for constitutional syphilis, but wanted first to see what

---

[7] Barker, L. F.: Oral Sepsis and the Digestive Apparatus. South. M. J. 7: 481 (July) 1918.

would happen after the removal of these septic roots, before going on with the treatment for syphilis. The result was most happy. I showed this patient to the Section on Ophthalmology at the New York Acadamy of Medicine, November 19, 1917.

Case 3.—L. F. C., aged 37, sent to me by Dr. H. H. Forbes, July 27, 1917, was complaining of failing vision in the right eye. With correction, he had vision: O. D. 20/200; O. S. 20/20. On examination, I found that he had an exudate choroiditis and large floating opacities in the vitreous of the right eye. His tonsils had been removed by Dr. Forbes. On examining his mouth, I found his gums very much inflamed, and on the right side, at the root of one of the molars, there was an abscess. There was also a bridge in his mouth. I advised an immediate roentgenogram of his teeth, which showed for one thing, that there was an old root in the gum of one of the central incisors, which had been pulled long before; also that there was an abscess cavity at the root of two of the upper molars on the right side. The root of the incisor was extracted; also two molars were pulled, and the bridge was removed. All of this work was done in the summer of 1917. The patient's Wassermann test and urine were negative and he had no internal treatment. The man had been improving ever since the dental work was done; July 25, 1918, he came to me with a vision in the right eye of 20/20 with correction.

Any part of the eye may be attacked as a result of dental infection, but by far the greatest number of cases show the iris, ciliary body, choroid or cornea to be affected.

Lewis [8] reports two cases of retinal hemorrhage due to bacterial toxins, and sets forth a new conception, namely, that in these cases there occurs an absorption into the blood of a soluble protein poison usually given off from focal infections, which has a solvent effect on the inter-

---

[8] Lewis, F. P.: A Bacterial Toxin as the Cause of Retinal Hemorrhage, J. A. M. A. 70: 1813 (June) 1918.

cellular cement substance of the cell wall, and that the hemorrhage is not due to alteration of the blood pressure, but to local lysis in the wall itself. It seems to me that Lewis' theory of this new pathology is a correct one, and will in the future explain many obscure cases of recurrent retinal hemorrhages which, in the past, have been diagnosticated as due to tuberculosis.

In operations on the eyeball when pyorrhea alveolaris is present, or when I suspect any trouble with the teeth, I always begin by referring the patients to an up-to-date dentist, who at once starts his treatment. When the dentist has finished with the patient, I generally wait from four to six weeks before operating, so as to try to eliminate all toxins from the system. Even in glaucoma I wait, if it is possible, to eliminate the three T's. All of this may seem heroic, but let me assure you that we are dealing with an arch enemy not to be trifled with, and my success in treating patients along these lines impels me to go forward.

### THE WAY TO "GET RIGHT" ON THE DENTAL QUESTION

To my mind, the way to get at the dental problem correctly is to start with children in the schools, as complete and proper mastication can not take place unless the mouth is in a healthy condition. Education along dental lines and in dental hygiene is just as necessary as education of the mind. Children who never use a toothbrush are constantly coming to my clinic. All they get here in our public schools in the way of dental hygiene is what they call "the toothbrush drill." What we really need here in New York and elsewhere are dental hygienists or dental dressers, to inspect the mouths of these school children regularly and systematically—to detect irregularities of the teeth; to detect cavities by probe and mirror; to chart defects correctly; to select temporary teeth for extraction; to clean deposits from the teeth; to extract the temporary teeth, and to apply disinfectants to the gums when inflamed, and above all, to educate the children in "dental hygiene." Women who do this work are given a nine months' course at Columbia University; then they stand an examination and are given a diploma as "dental hygienists." These dental dressers are, of course, to work under the supervision of some up-to-date dentist who can inspect their work from time to time. In this way the city would not be put to any great amount of expense, and the children would be well taken care of and receive scientific treatment. This work is all the more important when we stop to consider that at least 70 per cent. of our school children have defective teeth, that all defective teeth are injurious to health, and that some of them are a deadly menace to their owners.

### EYESIGHT OF SCHOOL CHILDREN

The dental dressers could also be taught to test the vision of all school children regularly, and those with defective eyesight could be referred to the clinics, as nothing handicaps a child's progress in education more than poor eyesight. Hints and pamphlets on the care of the eyes and the teeth should be published for distribution among the school children and the mothers. The pamphlets could also outline the diet for these children, and call the attention of the mothers to the harmful effects of sugars and candies.

The publishing of the pamphlets, the issuing of charts to be hung in the schoolrooms, and the preparing of exhibits to be used in the schools are some of the things which the dental dresser could do.

These suggestions of mine are all educational, and should be a part of the regular course of every child from the kindergarten until the day of graduation.

Diseases of middle life are fast increasing. They are microbic, of a chronic, recurring character, and are carried into the blood stream from a few foci, the mouth being the source of the greatest danger.

### THE TONSILS

The tonsils, on account of their position and structure, are more prone to infection than almost any other organ in the body. Because of their position, they come in contact with the various species of bacteria in the mouth cavity. More important than this, however, is the deposition of these bacteria with the food, in the lumina of the crypts. They therefore receive and transmit in many cases some infectious process.

Ziegler[9] reported a case of marked papilledena in a child, which was persistent for over a year, and which subsided and cleared up after the removal of the tonsils. This septic condition of the tonsils was well illustrated in a case of a physician which King[10] reported. The patient had arthritis and heart and kidney trouble with episcleritis. He was unable to attend to his professional duties. The whole systemic involvement was cleared up by the use of vaccines and the enucleation of the tonsils. I had the pleasure of seeing this physician eighteen months after operation. He had resumed his practice and had had no recurrence of any of his old troubles. He had gained in weight and was feeling fine.

When the tonsils are involved, anything less than the radical treatment is of little value. This was thoroughly demonstrated by Case 5.

Case 5.—Miss C. R., who first came to me, July 5, 1918, complained of "red eyes," as she called it, and had been under treatment for several years by two competent oculists. There was at this time marked redness and congestion of the bulbar conjunctiva, with some edema and some follicles on the palpebral conjunctiva. I examined the patient's eyes for refraction, and found that her error had been properly corrected. Looking her over for the

---

[9] Ziegler, S. L.: The Chemistry of Metabolism in Its Relation to Ocular Diseases, Ann. Ophth. (April, 1911), p. 250.

[10] King, J. J.: Further Observations on the Conellan-King Diplococcus Throat Infections, J. A. M. A. 68: 91 (Jan. 13) 1917.

three T's, I found that her teeth were excluded, but on examining her throat, it developed that she had two large, juicy tonsils which projected way beyond the pillars of the fauces. She was told that nothing could be done for her until her tonsils were removed. That day, the first time I had ever seen her, she was sent to consult a nose and throat specialist, and her tonsils were enucleated the next day. Her progress has been very marked ever since the operation, and now, six months afterward, "the red eyes" have disappeared, and she no longer has any chronic conjunctivitis. She remarked to me, "You are the first doctor who ever examined my tonsils." Think of it, a woman 26 years old with a low grade conjunctivitis, which had extended over a period of several years, who had never had her throat examined!

Osborne [11] thinks that a very large proportion of thyroid disturbance is caused by mouth and throat infections.

Recently Gardiner [12] has called attention to the fact that he has seen two typical cases of exophthalmic goiter which were permanently cured after one year's observation with the rest cure and the enucleation of the diseased tonsils. He also states that Morse was able to cultivate *Streptococcus viridans* from the thyroid glands removed from two typical cases of goiter.

Errors of diet and pyorrhea alveolaris are the starting points of nearly all cases of hyperacidity, hypoacidity and toxemia of the intestinal tract. How often is the dietetic condition discussed with our patients unless they have some obscure eye disease? How often do we demand of new patients coming to our offices an examination of the urine? I hold that every patient should be examined for the three T's and along these lines. How many of us warn patients against the excessive use of cane sugars,

---

[11] Osborne, O. T.: Tooth Infections, New York M. J. 107: 385 (March 2) 1918.

[12] Gardiner, H. C.: The Medical Treatment of Graves' Disease, New York State, J. M. (July) 1918, p. 267.

glucose and carbohydrates? When we stop to consider that after the Revolutionary War, the estimated consumption of sugar in the United States was only 7½ pounds per capita per annum, whereas in 1914, the per capita used amounted to about 90 pounds, as Joslin[13] says, "The marked increase in the consumption of sugar is appalling." He continues: "Since 1900 it has increased 17 per cent., and the mortality from diabetes mellitus has doubled in that time. Such a marked alteration in the diet of the nation is noteworthy and deserves attention."

It is in these civilized food products that we must look for the agents that are responsible for auto-intoxications from which the human race now suffers. The carbohydrates taken as sugars and starches are digested by the ptyalin of the saliva, and amylopsin, maltase and lactase of the pancreas, and the invertin of the intestinal juice, so that they are all reduced to a state of monosaccharids. Some of them are further broken up by bacteria,[14] with the formation of lactic acid, alcohol, marsh gas, hydrogen and butyric acid. In other words, there is nothing in the human stomach to render it physiologically assimilable. It is attacked there by bacteria which oxidize it and convert it into highly irritable organic acids and several alcohols. If taken in solution on an empty stomach, or when the food is passing into the small intestine, it becomes a food product, as the inverted ferments of the intestine break it up into glucose and levulose sugars, which are assimilable. If taken, however, with food which must remain in the stomach one or more hours, then sucrose becomes a foreign body, is acted on by bacteria, and its ultimate products become important factors in the production of our metabolic diseases.[15]

---

[13] Joslin, E. P.: The Treatment of Diabetes Mellitus, Ed. 2, p. 59.

[14] Matthews, A. P.: Physiological Chemistry, Ed. 2, p. 446.

[15] Deeks, W. E.: Gastric and Duodenal Ulcers, New York, M. J., Nov. 30, 1912, p. 1103.

As Hawk[16] says, "Chemically considered, the carbohydrates are aldehyd or ketone derivatives of complex alcohols." I think two things, therefore, are necessary for sugar fermentation: undigested sugar free in the stomach or intestine, and the bacteria in sufficient quantity to attack it. Overfeeding with sugar either given as too much food as a whole, or as too high sugar percentage, will cause fermentation. We must bear in mind that the gastric juice is not only a secretion but also an excretion, and anything that goes into the stomach that will produce fermentation will generally produce hyperacidity and will have a tendency to give us the acid stools.

Case 7.—A man, aged 20, came to my clinic with vision O. D., 20/20; O. S., 20/200, complaining of poor vision in the left eye. There was a circumcorneal injection of blood vessels of the left eye. The cornea was hazy, with few spots on Descemet's membrane, and the pupil was contracted. One could see that the eye was in a state of low grade inflammation. The teeth were negative as far as we could make out, although a roentgenogram of them was ordered. A Wassermann test was also taken. The tonsils had been removed when he was a boy. On getting his personal history, it developed that he had eaten about half a pound of chocolate every day for the last six months. He was a helper on an express wagon and visited many candy stores. He was taken off the sweets and given some atropin and hot bathing for his eye. He continued with his work.

Two weeks later roentgenoscopy of the teeth and the Wassermann test were negative; the urine was also negative, except for + + + + indican. The patient continued to improve, and at the end of three months was entirely well, with a vision of 20/30. One year has now elapsed and he has had no return of his malady. There is no doubt that this patient was suffering from sugar poisoning.

---

[16] Hawk, P. B.: Practical Physiological Chemistry, Ed. 5, p. 19.

Dietitians are not all agreed as to the value of sugar. I hold and believe that it is a toxic substance, and that children, as well as adults, should be deprived of its use so far as possible.

We see the baneful effects of candy and sweets in children coming to us with eczematous keratoconjunctivitis, one of the commonest diseases of the eye. It is a common disease in more senses than one, for it is common among the common people. The sight of a dirty and slatternly mother dragging two or more children equally dirty and slatternly, with eyes tightly closed is, unfortunately, too frequent in our out-patient clinics, and forms a sad picture which is familiar to us all. To my mind, the phlyctena, which is characteristic of the disease, is the ocular manifestation of a toxemia arising from some focus situated somewhere in the body. It is interesting to note that all of the children have gastro-intestinal disturbances, and the great majority of them have indican in the urine. This toxemia of the gastro-intestinal tract is brought on by the excessive use of candy and sweets, and bad teeth. Some of these unfortunate children also have adenoids and tonsils which must be removed. I know that a great many observers claim that there is a good deal of evidence to show that a tuberculous focus exists somewhere in the body. I do not think that their reasoning is sound. I hold that the three T's play the important part in the etiology of this disease. When the cause of the trouble is removed and these children have local treatment for their eyes, they get well.

Hill [17] has found that the stools of breast-fed babies are normally quite strongly acid in reaction, owing to the large amount of sugar and fat in breast milk. In severe cases of sugar fermentation, Hill recommends alkali treatment. He also maintains that a baby can take relatively more sugar than an adult, as the assimilation limit is much

[17] Hill, L. W.: Sugar in Relation to Infant Feeding, Boston M. & S. J. 179: 1 (July 4) 1918.

higher in infancy. Case 8 is most interesting, as it clearly shows the results of sugar poisoning.

Case 8.—Mrs. A. G., aged 27, married, came to me October 1, 1918, complaining of her vision being fogged, and of being unable to read. She had been under my care for her eyes for several years, and her vision in the past had always been normal. Her vision at this time was 20/200 in each eye. The pupils reacted sluggishly to light. Pressure on the eyeballs gave her pain. Her fields for form were normal with a central color scotoma for red, extending from the blind spot to the fixation point. The scotoma was more or less circular in shape, and not oblong, as we generally see in toxic amblyopia due to tobacco poisoning. There was no contraction of the peripheral field. Both fundi were normal. The optic disks were not swollen or edematous. She gave a history of consuming large quantities of sweets in the form of candy—about half a pound a day—and of drinking at least three ice cream sodas a day. I had a chemical analysis made of the blood and the urine. She was given a dose of castor oil, which has great power to sweep the intestine clean, after which I ordered a strict diet, without any sugars or sweets, and she was given one bottle of Bulgarian bacilli three times a day before meals.

October 6th, the report on the blood was that it was normal except for high blood sugar, from 0.15 to 0.18 per cent. The urine showed high specific gravity, 1.027, with a positive reaction for sugar, and high indican reaction. I thought from the start that I was dealing with a case of hyperglycemia, and these tests were still more convincing.

The average glucose content of normal blood is about 0.1 per cent. This increase in the blood sugar was brought about by the increased ingestion of carbohydrates in the form of sweets, for the reason that after the ingestion of large amounts of sucrose or glucose, causing the assimilation limit to be exceeded, and alimentary glycosuria will arise.

November 1st, I saw the patient again, at which time her vision was 20/70 in both eyes. The treatment was continued. The urine was examined again for sugar, but was negative. The diet was continued.

The patient was seen from time to time, and on December 1st her vision was normal again and she could read Jaeger No. 1.

This case is undoubtedly one of auto-toxemic amblyopia, due to the anomalies of metabolism. The opportunity was afforded for the production of toxic substances through the instrumentality of the sweets. Most candies contain inverted sugars and commercial "glucose," which are readily fermented either by bacterial action or by means of enzymes or activating bodies or agents. It is interesting to note right here that two-fifths of all the sugar that is consumed is eaten in the form of candy. The most frequent occurring examples of such a disturbance of vision is found in diabetes mellitus, in which we have constantly high blood sugar.

I hold that every case of retrobulbar neuritis should have a chemical analysis of blood and urine, even if the patient smokes and drinks. Estimations of the blood sugar are, undoubtedly, of great value in the treatment of these cases of toxic amblyopia.

There are two ways in which the sight may become affected in toxic amblyopia—endogenous and exogenous: the endogenous toxins are found in connection with hyperglycemia, diabetes mellitus, uremia, the puerperal state, or a septic process somewhere in the alimentary canal, while exogenous toxins are tobacco, ethyl or methyl alcohol, and numerous other substances.

Collins and Mayou state that the poison either acts on the ganglion cells of the retina or their synapses, or by the production of ischemia which interferes with their nutrition.

It is interesting to note also that Adams,[18] of Oxford, in speaking of twenty-nine acute cases of retrobulbar neuritis, states that two-thirds of these were in females between 15 and 30 years of age.

It is interesting to note that a substance which has been occasionally found in the lens, and which does not occur under normal conditions, is sugar.[19] Its presence in the lens in cases of diabetic cataract has attracted the greatest attention. Professor Kuhne was able to demonstrate it. It is also interesting to note that Lohmeyer[20] succeeded in demonstrating the presence of sugar in the vitreous taken from two human beings who had just died of diabetes. Deutschmann[21] proves the presence of sugar in the aqueous and vitreous of the body of a diabetic whose lens also contained sugar. This work by these investigators is most timely and most interesting, and only goes to show that sugar can leave the blood stream and may attack any of the tissues of the eye.

### HOW BAD TEETH AFFECT THE ALIMENTARY CANAL

Pyorrhea alveolaris, or Riggs' disease, is not only a serious condition, but also is very prevalent. This chronic disease loosens the teeth in the gums, or rather strips the gums from the teeth, so that they drop out, and mastication becomes impossible. But even more disastrous to health than the inability to masticate properly are the effects of the continued ingestion of toxic material which the pyogenic germs have developed. This toxic material, and bacteria mixed with the food, pass into the alimentary tract and bring about gastro-intestinal auto-intoxication which, in turn, is responsible for a large number of grave conditions. This is one reason why the teeth should be thoroughly investigated in every patient coming to us,

---

18 Adams, P. H.: Some Points in Retrobulbar Neuritis, with Special Reference to Prognosis, Brit. J. Ophth. 2: 522 (Oct.) 1918.

19 Stricker, L.: The Crystalline Lens System, 1898, p. 67.

20 Lohmeyer: Beitrag zur Histologie und Aetiologie der Erworbenen Linsenstaare, Ztschr. f. rat. Med. New Series, p. 99.

21 Deutschmann: Zur Regeneration des Humor aquosus, Arch. f. Ophth. 26: 99.

whether we suspect a septic process in the alimentary canal or not, as pus may be swallowed with the food over long periods, or metastatic infections may occur. Barker says that some cases of gastric and duodenal ulcers are secondary to oral sepsis.

Of course, the examination of the urine of all new patients coming to the office is imperative. It is also essential to have the indican content of the urine. This gives a rough index of the extent of the putrefactive process in the intestine. It is therefore the duty of every physician, whether he be specialist, general practitioner or surgeon, to examine the urine of all new patients coming to his office, and to demand a urinalysis of all old patients once a year. I can not lay too much stress on the importance of having the urine of all patients examined, and I think that the failure on the part of the physician to do this is simply inexcusable.

Joslin says that "the time is not far distant when the progressive dentist, even, will demand a urinary report of all his patients." I am convinced that the indican reaction is passed over too lightly by physicians in general. Soper [22] says: "I have often noted the good effects produced in certain cases of toxic headaches by sharply limiting the protein and reducing or omitting sugar from the dietary. Patients of this class usually present excessive indicanuria, much colonic flatus, froth and fermentative feces. Moreover, they are in the habit of consuming large quantities of cane sugar."

Apropos of the carbohydrate element, the work of Pemberton [23] is especially interesting. He produced good results in cases of rheumatoid arthritis by a radical reduction of carbohydrate in the dietary.

---

[22] Soper, H. W.: Auto-intoxication in Chronic Constipation, J. A. M. A. 69: 1511 (Nov. 30) 1917.

[23] Pemberton, R.: Metabolism and Rheumatoid Arthritis, Am. J. M. Sc. 151: 351 (March) 1916.

Hagelberg[24] found an abnormally high sugar content in the blood in twenty-six cases of arteriosclerosis. A thorough examination of urine and feces, and a chemical blood examination, will give us a reliable index of the putrefactive condition in the intestinal tract.

Why the optic nerve should be affected in some cases of gastro-intestinal auto-intoxication, the uvea in other cases, the cornea in some and the vitreous in still others, I am not able to state, nor am I able to state just how the conditions are brought about. We must all admit, however, that toxemia may result from chemical changes in the intestinal contents, and that absorption of protein toxins from the intestinal tract does take place, and that the blood stream is the carrier of the infection, the three common foci being the teeth, tonsils and the intestinal tract.

SUMMARY AND CONCLUSIONS

*The Teeth.*—1. If the teeth are properly treated from the outset, they will not abscess. If we do our part nature will do hers. That is the reason why we must start with the children in the schools.

2. These suggestions on my part are educational, and should be a part of the regular course of every child, from the kindergarten age until the day of graduation.

3. I strongly urge the country to "get right" on the dental question.

5. Modern dentistry is relieving the world of much misery, by the watchful care of the foci connected with the teeth, and the time is not far distant when modern dentistry will be made a department of medicine.

7. While many of the results of oral infections are apparent to the eye, the deep-seated and hidden foci, which

[24] Hagelberg, M.: Berl. klin. Wchnschr. 39: 1877 (Sept. 30) 1912; abstr. J. A. M. A. 59: 1753 (Nov. 9) 1912.

are frequently the most virulent, are entirely hidden and can only be revealed by the roentgenogram.

8. Diseases of middle life are fast increasing, and to my mind the mouth and the diet are the sources of the greatest danger.

9. As complete and proper mastication can not take place unless the mouth is in a healthy condition, I hold that a dirty mouth is one of the greatest menaces of the human race.

*The Tonsils.*—1. The tonsils receive and transmit, in many cases, some infectious process. Because of their position and structure, the tonsils are more prone to infection than many others organs in the body. Because of their position, they come in contact with various species of bacteria found in the mouth. More important than this, however, is the deposition of these bacteria with the food which we find in the lumina of crypts.

2. It has been demonstrated that the tonsils are the principal foci of infection in the throat carriers of hemolytic streptococci.

3. All patients coming to the office should be examined for diseased tonsils.

4. When the tonsils are diseased, anything less than the radical treatment is of little value, as they are carriers of infection.

*The Alimentary Canal.*—1. Focal infections, especially those of the teeth, tonsils and alimentary canal, to use the words of Professor Osborne, of Yale, are ''no fad,'' but realities to be looked for and eradicated, if we are to cure our patients.

2. Errors of diet and pyorrhea alveolaris are the starting points of nearly all cases of hyperacidity, hypoacidity and toxemias of the intestinal tract. Children are often improperly fed, with the result that about 70 per cent. of school children have dental cavities; and when decay sets in early, the teeth become septic, poisoning the food eaten, so that systemic infection is frequent. Decayed teeth afford a ready passage into the system for disease germs. Large numbers of children have tuberculous glands which, in my opinion, are incident to such foci.

3. Sugar, chemically, is a solid alcohol. From a local little used product it has become the universal condiment in the food of the civilized races. It is used in man's drink, in his food and as his refreshment between meals, not only in solution, but also in the form of the ubiquitous candies of commerce. We must also bear in mind that two-fifths of all the sugar consumed is eaten in the form of candy.

4. Dietitians are not all agreed as to the value of sugars. I hold and believe that they are toxic substances, and that children as well as adults should be deprived of their use as far as possibble, as they are chemical alcohols. When the hydrochloric acid of the gastric juice is diminished in quantity (hypoacidity), as it may be in many cases of functional or organic disease, there is no check to the growth of the micro-organisms in the stomach; then the sugars are acted on by the bacteria, and fermentation takes place, with the formation of large amounts of such substances as lactic acid and butyric acid; in some cases a pyloric spasm is produced, thereby more markedly increasing fermentation and acidity with their end-product results. Hawk says that there are certain of the more resistant spores which even the normal acidity of the gastric juice will not destroy.

5. It is difficult for me to avoid the conclusion that the unrestricted use of all carbohydrates must be deleterious to the human economy.

6. If the sugar poison can invade the aqueous, the vitreous and the nutritive stream of the lens, then why can it not produce an inflammatory process in any of the tissues of the eye? On account of the insidious onset, the cause of the trouble is liable to escape recognition unless the dietetic condition is kept well in mind.

7. We must educate our patients to look after their diet, teeth and tonsils, and impress on them the great importance of having their urine examined once a year. In obscure conditions of the eye, we must go a step farther and have the feces of the patient examined, to see if the stools are toxic, and last but not least, to insist on a chemical examination of the blood, as a thorough investigation of the urine, feces and blood will give us a reliable index of the putrefactive condition of the intestinal tract.—*Journal A. M. A.*, October 11, 1919.

